# Genomic analysis of invasive and non-invasive disease-causing Streptococcus pneumoniae isolated from children between 2014 and 2023 in Suzhou, China

**DOI:** 10.1099/mgen.0.001398

**Published:** 2025-06-02

**Authors:** Lili Huang, Alannah C. King, Yue Liu, Harry C. H. Hung, Stephen D. Bentley, Yue Jiang, Panpan Lv, Xuebin Xu, Stephanie W. Lo, Mingliang Chen

**Affiliations:** 1 Laboratory Department, Children’s Hospital of Soochow University, Suzhou, Jiangsu, PR China; 2 Parasites and Microbes, The Wellcome Sanger Institute, Wellcome Genome Campus, Hinxton, Cambridge, UK; 3 Department of Microbiology, Shanghai Municipal Center for Disease Control and Prevention, Shanghai, PR China; 4 Research and Translational Laboratory of Acute Injury and Secondary Infection and Department of Laboratory Medicine, Minhang Hospital, Fudan University, Shanghai, PR China; 5 Milner Centre for Evolution, Department of Life Sciences, University of Bath, Bath, UK; 6 The Great Ormond Street Institute of Child Health, University College London, London, UK

**Keywords:** Global Pneumococcal Sequencing project, invasive, multidrug resistance, pneumococcal conjugate vaccine (PCV), *Streptococcus pneumoniae*

## Abstract

In 2017, 553,000 clinical cases of *Streptococcus pneumoniae* in children were reported in China, although the pneumococcal conjugate vaccine (PCV), which targets the pneumococcal capsule, is not included in the Chinese National Immunization Program (NIP) for children. Therefore, the PCV uptake rate is very low. To investigate the *S. pneumoniae* population over the past 10 years in China, we collected 418 S. *pneumoniae* isolates from children with pneumococcal diseases in Suzhou, China, 2014–2023, and whole-genome sequenced them. A total of 27 serotypes expressed by 36 Global Pneumococcal Sequence Clusters (GPSCs) that encompassed 72 sequence types were identified, with serotype 19F (38.3%, *n*=160) and GPSC1 (60.8%, *n*=254) as the predominant serotype and lineage, respectively. We found that the majority (64.8%, *n*=271) of samples represented serotypes that are covered by the GSK 10-valent PCV (PCV10) formulation and that even more were covered by the SII PCV10 formulation (89.2%, *n*=373). Almost all (94.3%, *n*=394) samples represented serotypes that are included within the 13-valent PCV (PCV13) vaccine formulation. This suggests that the inclusion of the PCV in the NIP would lead to significant benefits for child health. Also, we observed that no significant differences were seen in the serotypes or lineages in cases of invasive and non-invasive pneumococcal diseases.Additionally, we investigated the prevalence of antimicrobial resistance within the population and found that 99.8% (*n*=417) of isolates were predicted to be resistant to at least one antibiotic tested. This again supports the need to increase PCV uptake to prevent infections with antibiotic-resistant *S. pneumoniae* and to reduce the number of infections in general, consequently lowering the consumption of antibiotics. In summary, the PCV13 vaccine could potentially cover over 90% of invasive and non-invasive *S. pneumoniae* isolates in Suzhou, China. Therefore, increasing the uptake of PCVs by including PCV13 in the NIP would lead to significant benefits for child health.

­

**Impact Statement**

Despite high levels of pneumococcal disease, the pneumococcal conjugate vaccine (PCV) is not part of the Chinese National Immunization Program (NIP). This study suggests that including PCV is likely to reduce the prevalence of the pneumococcal serotypes associated with disease in Suzhou, China. Additionally, this study raises awareness of the exceptionally high levels of antimicrobial resistance (AMR) within the Suzhou pneumococcal population, which represents a significant public health risk. The introduction of PCV in NIP, alongside the implementation of antibiotic stewardship, has a strong potential to reduce AMR.

## Data Summary

Genome sequences are deposited in the GenBank (Bioproject, PRJNA1154805); the accession number and a phylogenetic snapshot are available at https://microreact.org/project/gps2-china. Data can also be found and downloaded through Monocle (https://data-viewer.monocle.sanger.ac.uk/project/gps). Metadata of the pneumococcal isolates in this study is submitted as a supplementary file. The authors confirm that all supporting data, code and protocols have been provided within the article or through supplementary data files.

## Introduction

*Streptococcus pneumoniae* (pneumococcus) is a leading vaccine-preventable cause of childhood diseases. This disease can be classed as invasive pneumococcal disease (IPD), where pneumococcal bacteria can be cultured from a usually sterile site [1], or non-invasive pneumococcal disease, such as otitis media or pneumonia. It was estimated that nearly nine million pneumococcal disease cases occurred in children aged under 5 years old worldwide in 2015 and that the pneumococcus was responsible for 317,000 deaths [2]. In China, *S. pneumoniae* is the predominant bacterium responsible for bacterial infections in children. The Infectious Disease Surveillance of Paediatrics data show that it is the leading bacterium isolated from lower respiratory tract samples, accounting for 22.5% of clinical cases [3]. In 2017, China reported 553,000 clinical pneumococcal cases in children, with 218,200 severe cases, including 8,000 deaths [4]. Therefore, *S. pneumoniae* remains a significant cause of invasive diseases in children under 5 years old. Despite preventive vaccines, pneumococcal diseases continue to be a substantial health burden, with China reporting thousands of severe cases and deaths annually [1,2].

Pneumococcal conjugate vaccines (PCVs) have proved to be effective in reducing childhood IPD in many countries [5,10]. However, despite 13-valent PCV (PCV13) being licensed for use in 2016, PCV vaccinations are not part of the Chinese National Immunization Program (NIP), and the administration of PCVs largely relies on personal payment and voluntary vaccination. Completing the full vaccination course can cost between 2,400 to 2,800 RMB (~$330–385 USD), making it a significant financial burden for many families [11]. Due to the high cost of PCVs and a lack of public awareness regarding their necessity and importance, the PCV vaccination rate in China remains low. Even in economically developed areas like Shanghai, the PCV13 vaccination rate is only 10.2%, whilst in less developed Western regions, the vaccination rate is below 1% [3]. There are three main PCV formulations available in the market at the time of writing. Two are ten-valent vaccines: PCV10 (GSK) contains the serotypes 1, 4, 5, 6B, 7F, 9V, 14, 18C, 19F and 23F, whilst PCV10 (SII) contains the serotypes 1, 5, 6A, 6B, 7F, 9V, 14, 19A, 19F and 23F. Finally, the PCV13 includes all serotypes in both PCV10 formulations alongside serotype 3.

The city of Suzhou, located in Jiangsu province in eastern China, has a population exceeding 10 million and is one of the most economically developed cities in the country. Jiangsu province has a very low PCV vaccination rate of 1.7% [3]. The Children’s Hospital of Soochow University is a prominent paediatric tertiary hospital in the Suzhou region, featuring 1,500 beds. Annually, the hospital manages over 3 million outpatients and admits more than 80,000 inpatients.

Antibiotic consumption in China is very high due to a variety of factors, including patient pressure, financial incentives and a lack of knowledge amongst physicians when prescribing antibiotics, and this has led to generally very high rates of antimicrobial resistance (AMR) [12]. Across China, *S. pneumoniae* has been seen to exhibit significant resistance to multiple antibiotics, particularly clindamycin (CLI), erythromycin (ERY) and tetracycline (TET), with resistance rates exceeding 94% [13]. During 2006–2016, 0.7%–7.4% of isolates from children’s respiratory samples are non-susceptible to penicillin (PEN) [11,13,15]. Studies in children from Suzhou show that the non-susceptibility rates to common first-line treatments for respiratory infections, such as PEN, amoxicillin (AMO) and cefotaxime (TAX), are 9.5%, 27.7% and 27.2%, respectively [16].

In this study, we undertook whole-genome sequencing on a collection of 418 disease-causing pneumococcal isolates collected from young children in Suzhou, China, between 2014 and 2023 as part of the Global Pneumococcal Sequencing project [17]. We aimed to update knowledge on the current lineages, serotypes and antibiotic resistance of *S. pneumoniae* circulating in the city of Suzhou, Jiangsu province, China. The data generated will provide evidence to support future prevention of pneumococcal disease and treatment strategies, including the inclusion of the PCV13 vaccine in the NIP.

## Methods

### Bacterial culture and identification

All patients with infections caused by *S. pneumoniae* at the Children’s Hospital of Soochow University from 2014 to 2023 were enrolled in this study. All isolates in this study were cultured from clinical specimens obtained from children under 16 years old. Pus and tissue were plated on Columbia blood agar plates. Blood and cerebrospinal fluid specimens were cultured using the BD BACTEC™ FX blood culture system, and positive samples were transferred to Columbia blood agar plates. The plates were incubated with 5% CO_2_ at 37 °C for 18–24 h. The colonies that showed α-haemolysis on the plates were further identified using Optochin disc sensitivity. For an ambiguous result on Optochin, the bile soluble test was performed to confirm the result [18]. All the suspected isolates were also tested using MALDI-TOF mass spectrometry (bioMérieux). All the pneumococcal isolates were stored in 10% skim milk at −80 °C.

## Isolates metadata

A total of 418 *S*. *pneumoniae* isolates were analysed in this study. A total of 95.0% (*n*=397/418) of samples were from children aged 5 years or under. The remaining samples (*n*=21) were from children between the ages of 6 and 15 years old. The clinical manifestations observed were otitis media (*n*=244), meningitis (*n*=71), sepsis (*n*=55), pneumonia (*n*=18), bacteraemia (*n*=12), bronchitis (*n*=7), urinary tract infection (*n*=1) and other (*n*=9). There was one recorded case of both pneumonia and bacteraemia. Among the 418 isolates, 139 isolates were identified as IPD strains, 271 isolates as non-IPD strains and 8 isolates were categorized as unknown.

## Genome sequencing and analysis

The pneumococcal isolates were sequenced on an Illumina HiSeq platform to produce paired-end reads of 150 bp in length, and the assembled sequences were deposited to the GenBank (Data S1, available in the online Supplementary Material). Whole-genome sequencing (WGS) data were processed as previously described [19]. Briefly, we inferred serotype and resistance for 16 antibiotics, including PEN, AMO, meropenem (MER), TAX, ceftriaxone (CFT), cefuroxime (CFX), ERY, CLI, quinupristin-dalfopristin, linezolid, cotrimoxazole (COT), TET, levofloxacin, chloramphenicol (CHL), rifampin, doxycycline (DOX) and vancomycin from the genomic data using SeroBA (version 1.0.0) [17] and a resistance detection pipeline developed by Centers for Disease Control and Prevention (CDC), respectively [20,24]. Vaccine serotypes (VT) were defined by serotypes in the PCV13 (Pfizer), including 1, 3, 4, 5, 6A, 6B, 7F, 9V, 14, 18C, 19A, 19F, 23F, and other serotypes were grouped as non-VT (NVT). The predicted minimum inhibitory concentration (MIC) values were interpreted according to Clinical Laboratory Standards Institute breakpoints [25], M100-S34). PEN resistance was defined as MIC of ≥0.12 µg ml^−1^, according to meningitis breakpoints. Our previous study showed high concordance between phenotypic and genotypic results of serotypes and antibiotic resistance [26]. Among these antibiotics, the 12 commonly used antibiotics AMO, CFT, TAX, CFX, MER, PEN, CHL, CLI, COT, DOX, ERY and TET were used for resistant pattern analysis. Pneumococcal lineage was defined by assigning a Global Pneumococcal Sequence Cluster (GPSC) to each isolate using PopPUNK (version 2.6.0) [27] and v6 of the PopPUNK reference database of pneumococcal isolates [28]. In addition, the multilocus sequence type was derived from the genome data using MLSTcheck (version 2.1.1706216) [29]. Phylogenetic analysis was performed on all isolates by constructing a maximum likelihood tree using FastTree (version 2.1.10) [30] based on SNPs extracted from an alignment generated by mapping reads to the reference genome of *S. pneumoniae* ATCC 700669 (NCBI accession no. FM211187) using Smalt (version 0.7.4; https://github.com/rcallahan/smalt). The metadata (Data S2) and analysis results can be interactively visualized online using the Microreact tool at https://microreact.org/project/gps2-china.

## Statistical analysis

For the purposes of comparing cases of IPD and non-IPD, the clinical manifestations ‘otitis media’, ‘eye discharge’, ‘pneumonia’, ‘urinary tract infection (UTI)’ and ‘bronchitis’ were considered non-IPD; ‘other’ and ‘joint fluid/pus’ were classed as unknown. The clinical manifestations ‘meningitis’, ‘sepsis’, ‘bacteremia’ and ‘pneumonia, bacteremia’ were categorized as IPD. Where we analysed vaccine types (VT) compared with non-vaccine types (NVTs), VTs were the serotypes present in the PCV13 formulation:1, 3, 4, 5, 6A, 6B, 7F, 9V, 14, 18C, 19A, 19F, and 23F. This encompasses serotypes in either of the PCV10 formulations.

Differences between the IPD samples and non-IPD samples were calculated using Fisher’s Exact test using a dataset that only included samples from pre-2018, when the sampling strategy changed. Before carrying out any Fisher’s Exact test, we calculated the number of samples that we need to achieve an 80% statistical power with a significant level of *P* value <0.05 using the R package pwr, which contains functions for basic power calculation [31]. Where we did not have enough samples to achieve 80% power, Fisher’s Exact test was not performed. When comparing the differences in serotype and GPSC distribution between IPD and non-IPD cases, Fisher’s Exact test was run in R with a simulated *P* value and 500,000 bootstraps due to the large number of variables. Multiple testing was adjusted using the Benjamini–Hochberg false discovery rate of 5%.

To analyse differences in AMR between IPD and non-IPD isolates, the dataset was filtered to only contain samples from between 2014 and 2017, as the sampling strategy changed in 2018 to focus on IPD cases. Fisher’s Exact test was performed, and multiple testing was adjusted using the Benjamini–Hochberg false discovery rate of 5%. Differences were considered significant if *P*<0.05.

Multidrug resistance was defined as non-susceptibility to at least three out of the five antibiotics, PEN, TET, ERY, CHL and COT.

The R script used for this analysis can be found at https://github.com/ak2022/GPS2_China.

## Results

### Data overview

We observed a noticeable decline in the number of collected isolates per year in the dataset since 2018, with a greater proportion of isolates being from IPD cases compared with non-IPD (Fig. S1). This reduction is attributed to the hospital’s increased focus on IPD cases in recent years. As a result, from 2018 onwards, the hospital has primarily collected isolates from blood and cerebrospinal fluid, leading to a significant drop in the overall number of isolates, and a greater proportion of recent isolates being from IPD cases (Fig. S1).

### Serotypes and PCV coverage

**Fig. 1.**
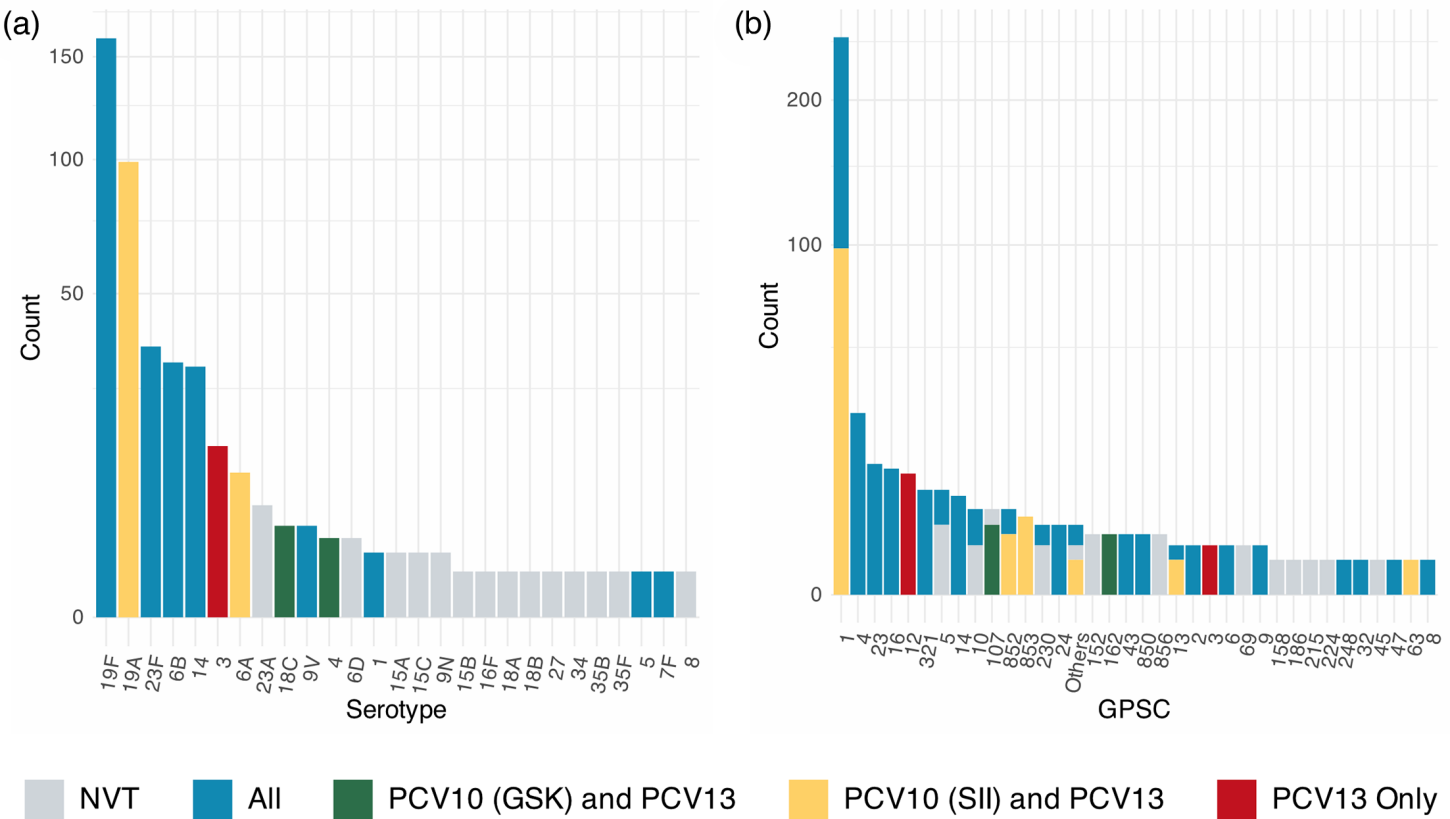
Distribution of serotypes (**a**) and GPSCs (**b**) within 418 Suzhou isolates, coloured by their coverage by the PCV10 (GSK), PCV10 (SII) or PCV13 vaccines. Only 5.7% of isolates within the dataset belonged to serotypes not covered by PCV10 (GSK), PCV10 (SII) or PCV13.

Serotypes predicted from WGS data revealed 27 serotypes expressed by 36 pneumococcal lineages (or GPSC) that encompassed 72 sequence types (Fig. 1). The five most prevalent serotypes were serotype 19F (38.3%, *n*=160), 19A (23.7%, *n*=99), 23F (8.4%, *n*=35), 6B (7.4%, *n*=31) and 14 (7.2%, *n*=30), and together they accounted for 85% of the whole collection. All of these major serotypes are covered by PCV10 (SII) and PCV13; however, serotype 19A is not covered by PCV10 (GSK). Across the whole dataset, we found PCV10 (GSK) to have 64.8% coverage, PCV10 (SII) to have 89.2% and PCV13 to have 94.3%. The large difference in coverage between PCV10 (GSK) and PCV10 (SII)/PCV13 is due to the lack of serotype 19A. PCV13, having the highest coverage, underscores the limited impact of the PCV13 vaccine in Suzhou currently due to the low uptake rate. Although the PCV13 vaccine was made available privately within China in 2016 [32], the low vaccination rate of 6.5% (2016–2018) [33] is leading to the vaccination having very little impact on the pneumococcal population in Suzhou, Jiangsu.

Although PCV13 showed the highest coverage of serotypes in the dataset, it was not 100%. Overall, only 5.7% (*n*=24) of samples within the dataset were NVT, with the largest number of non-PCV13 samples being serotype 23A (25.0%, *n*=6, four expressed by GPSC5 and two expressed by GPSC10) or 6D (12.5%, *n*=3, all GPSC856). Neither of these is included in the PCV15 or PCV20 formulations. Other non-PCV13 serotypes were 9 N (*n*=2), 15C (*n*=2), 15A (*n*=2), 18B (*n*=1), 15B (*n*=1), 34 (*n*=1), 35B (*n*=1), 18A (*n*=1), 16F (*n*=1), 27 (*n*=1) and 8 (*n*=1). None of these are within the PCV15 formulation, but serotypes 15B and 8 are within the PCV20 formulation. This suggests that despite the introduction of higher valency vaccinations to market, these vaccinations may have marginal benefits compared with PCV13. It is important to monitor these serotypes and lineages, as they are likely to persist even in a population with high PCV13 coverage.

### Pneumococcal lineages

Among the 418 isolates, the 5 most predominant pneumococcal lineages were GPSC1 (60.8%, *n*=254), GPSC4 (6.5%, *n*=27), GPSC23 (3.3%, *n*=14), GPSC16 (3.1%, *n*=13) and GPSC12 (2.9%, *n*=12), which together accounted for 77% (320/418) of the collection (Fig. 1). All major lineages express serotypes found within PCV13.

There were four lineages expressing a combination of PCV13 VT and NVT: GPSC5 (*n*=9), GPSC10 (*n*=6), GPSC107 (*n*=6) and GPSC230 (*n*=4). The largest was GPSC5, which expressed NVT serotype 23A (44.4%, *n*=4) alongside VTs 19F (22.2%, *n*=2) and 23F (33.3%, *n*=3). Both GPSC5 and GPSC10 have been seen to be global-spreading lineages, with both of these lineages known to mediate serotype replacement [34,36]. We investigated a larger dataset to better understand these lineages in a global context (*n*=26,306, available on Monocle at https://data-viewer.monocle.sanger.ac.uk/project/gps, last access 3 March 2025). GPSC107 has only been identified in China (*n*=6) and Thailand (*n*=21), and the vast majority (98.3%, *n*=57/58) of GPSC230 isolates have been identified from Asian countries such as Nepal (44.8%, *n*=26), China (15.5%, *n*=9), India (15.5%, *n*=9) and Bangladesh (13.7%, *n*=8).

Eight GPSC lineages expressed only NVT serotypes. These were GPSC152, GPSC856, GPSC69, GPSC158, GPSC186, GPSC215, GPSC224 and GPSC45. Of these, only GPSC152, GPSC856 and GPSC69 were represented by more than a single sample. GPSC152 expressed serotypes 15C (66.7%, *n*=2) and 15B (33.3%, *n*=1). According to the global dataset, GPSC152 is only seen within China. Specifically, the GPSC152 isolates were detected in Hong Kong (76.0%, *n*=19), the Chongqing municipality (16.0%, *n*=4), the Beijing municipality (4.0%, *n*=1) and Gansu province (4.0%, *n*=1). Two GPSC152 samples have no regional data. All GPSC152 were multidrug-resistant. All these regions are geographically separated from each other, suggesting a wider existence of GPSC152 pneumococci across China, and that GPSC152 may represent a China-specific multidrug-resistant lineage. Similar to our observations in Suzhou, the GPSC152 isolates were mostly serotype 15B or 15C (92.6%, *n*=25), with two members expressing serotype 13 (7.4%, *n*=2). This reflects what is seen in the Suzhou dataset. GPSC856 expressed only serotype 6D in all samples (*n*=3). There was only one other sample of GPSC856 in the GPS1 database, identified in the Chongqing municipality of China and expressing NVT serotype 6D, in line with the Suzhou dataset. It is possible that GPSC856 also reflects a China-specific lineage; however, the small number of samples makes this difficult to confirm. In the Suzhou dataset, GPSC69 only expressed serotype 15A (*n*=2). In the GPS1 dataset, GPSC69 members were found in Peru (33.3%, *n*=15), China (22.2%, *n*=10) and Nepal (22.2%, *n*=10), expressing mostly serotype 15A, with a single isolate from Peru expressing serotype 19A. This suggests that GPSC69 is also a wide-spreading lineage.

These data suggest that the introduction of any PCV to the NIP would have the potential to reduce cases of pneumococcal disease. However, there are lineages present in the population that either express a mix of NVTs and VTs, or only NVTs. In particular, GPSC5 and GPSC10 have been reported to escape PCV13 protection in other countries [34,36]. It is likely that these populations will expand to fill the gap left after the introduction of a childhood PCV programme, so continuous monitoring would be beneficial.

### IPD and non-IPD manifestations

**Fig. 2.**
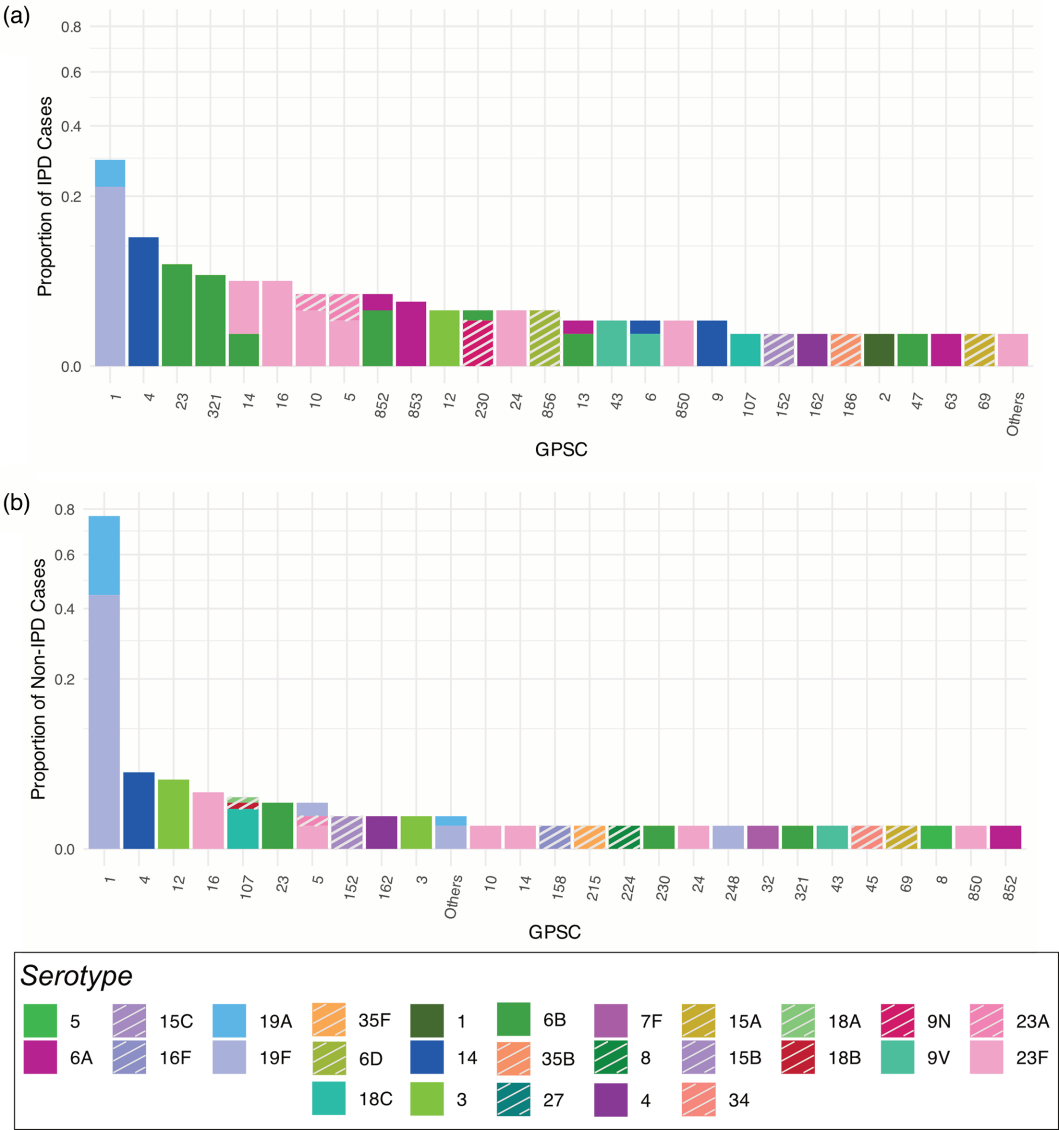
Distribution of GPSCs in IPD (**a**) and non-IPD (**b**) cases. There is no significant difference in the distribution of GPSCs between the two datasets (*P*=1), but a significant difference in serotypes (*P*=0.02). A greater percentage of non-IPD cases was caused by serotypes 19F and 19A compared with IPD cases (78.6% vs. 29.5%). NVTs are denoted with stripes.

We investigated the difference in serotype and lineage distribution stratified by IPD cases and non-IPD cases for samples collected before 2018 (Fig. 2). Interestingly, there were no statistically significant differences between the IPD cases and non-IPD cases from the perspective of GPSC lineage (*P*=1). However, there was a statistically significant difference from the perspective of serotype (*P*=0.02), with a much greater proportion of non-IPD cases being caused by serotypes 19F and 19A (Fig. 2). Both datasets had the greatest proportion of samples from the lineage GPSC1, and the dominant serotype was 19F.

### Antibiotic resistance

**Fig. 3.**
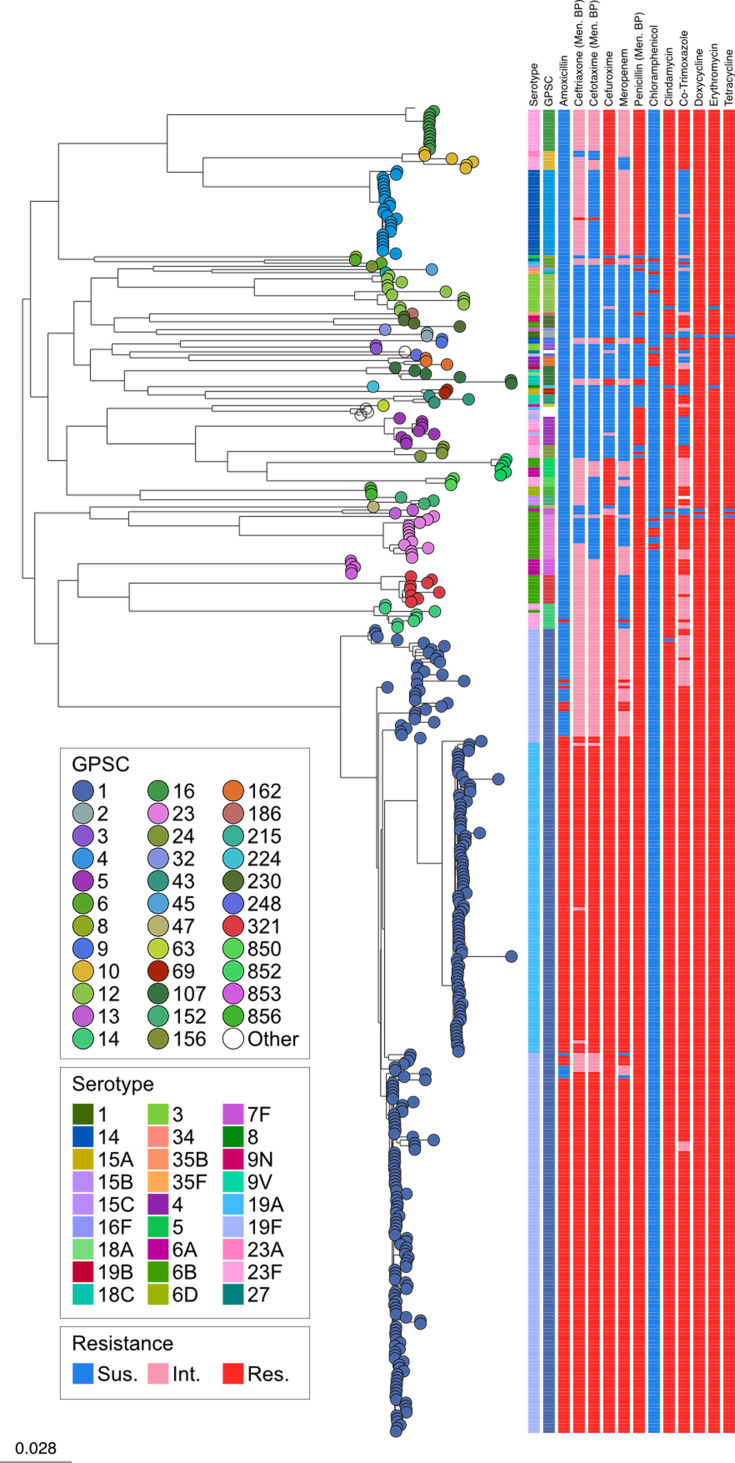
A maximum-likelihood SNP phylogeny of the 418 *S*. *pneumoniae* isolates from Suzhou, available at https://microreact.org/project/gps2-china. The predicted antibiotic resistance of samples within the Suzhou dataset is also shown. GPSC1, the largest lineage in the dataset, is also multi-drug resistant. Resistance for PEN, CFT and TAX was reported at the meningitis breakpoint.

Finally, we investigated the prevalence of predicted antibiotic resistance based on genomic sequence within the dataset (Table 1). Of the 418 isolates, 95.5% (*n*=399/418) were predicted to be multidrug-resistant, defined here as being non-susceptible to at least three of the antibiotics PEN, CHL, COT, TET or ERY (Fig. 3), including all samples within GPSC1, the largest lineage seen in the dataset. Only a single sample was predicted to be susceptible to all classes of antibiotics investigated.

**Table 1.** Antibiotic resistance across the samples from Suzhou, China

Antibiotic	Non-susceptible isolates			
	VT (*n*=394)		NVT (*n*=24)	
	Count	Percentage %	Count	Percentage %
Amoxicillin	221	56.1	0	0
Ceftriaxone (Men. BP*)	341	86.5	6	25.0
Cefotaxime (Men. BP)	305	77.4	0	0
Cefuroxime	353	89.6	9	37.5
Meropenem	316	80.2	2	8.3
Penicillin (Men. BP)	361	91.6	14	58.3
Chloramphenicol	13	3.3	1	4.2
Clindamycin	389	98.7	23	95.8
Cotrimoxazole	344	87.3	13	52.2
Doxycycline	391	99.2	24	100
Erythromycin	392	99.5	23	95.8
Tetracycline	391	99.2	24	100
MDR	380	96.4	19	79.2%

*Men, meningitis; BP, breakpoint based on Clinical and Laboratory Standards Institute Guidelines [M100-S34] [25]. MDR defined as non-susceptibility to at least three of penicillin, tetracycline, erythromycin, chloramphenicol and cotrimoxazole.

The non-susceptibility prevalence of the 418 *S. pneumoniae* isolates to various antibiotics from 2014 to 2023 indicated consistently high non-susceptibility rates for many antibiotics (Table S1), suggesting widespread resistance. Certain antibiotics, such as CLI, MER, DOX, ERY and TET, exhibited high non-susceptibility prevalence, often nearing or reaching 100% (Table 1). In particular, the high non-susceptibility rate of 76.1% to MER reflects concerns in a recent publication about high levels of carbapenem resistance in *S. pneumoniae* [37]. CHL was notable with very low non-susceptibility rates (3.3%). Once a common treatment for pneumococcal infections, CHL is now seldom used due to its potential for serious side effects, including severe bone marrow suppression and ‘gray baby syndrome’, particularly in children [38].

Earlier in this study, GPSCs containing NVTs were identified as they could expand in number after the introduction of the PCV vaccination. All four lineages that were seen to express a mix of VT and NVT serotypes (GPSC5, GPSC10, GPSC107 and GPSC230) were predicted to be multidrug resistant. Of the lineages that expressed only NVTs (GPSC152, GPSC856, GPSC69, GPSC158, GPSC186, GPSC215, GPSC224 and GPSC45), all were multidrug resistant aside from GPSC69, GPSC158 and GPSC224, which was predicted to exhibit resistance to both DOX and TET. All NVT members were susceptible to AMO and TAX. Additionally, VT isolates show significantly higher non-susceptibility prevalences across most antibiotics compared with NVT isolates.

Finally, we compared antibiotic resistance profiles between IPD and non-IPD isolates for each antibiotic. We found that AMO, CFT, TAX and MER all had significantly more resistant isolates in non-IPD cases than in IPD cases (*P*<0.05) (Tables S2 and S3).

## Discussion

This study provides valuable insights into the serotype distribution and genetic diversity of *S. pneumoniae* in Suzhou, China, from 2014 to 2023, shedding light on the epidemiology of pneumococcal disease in children. We identified 27 serotypes across 36 GPSC lineages, with GPSC1 being the predominant lineage, highlighting the variation in pneumococcal strains circulating in the region. We found the most prevalent serotypes to be 19F and 19A. These were identified as the most prevalent serotypes in samples collected from patients in West China Hospital between 2018 and 2022 [39] and were among the most prevalent serotypes in samples from community-acquired pneumonia patients and healthy asymptomatic participants in Sichuan [40]. The study suggests that the PCV13 vaccine could cover more than 90% of *S. pneumoniae* isolates linked to both invasive and non-invasive diseases in children in Suzhou, and so it would be a beneficial inclusion to the NIP. This aligns with the growing global consensus that expanding access to pneumococcal vaccines is a critical strategy for reducing the incidence of pneumococcal diseases in children [41].

Although our study suggests that the introduction of higher valency PCVs, such as PCV15 and PCV20, is unlikely to significantly impact the current pneumococcal population more than PCV13, they should not be ignored. However, these vaccines offer broader serotype coverage; continued surveillance is necessary to monitor the potential shift in the serotype distribution over time. The inclusion of higher valency PCVs in future vaccination programmes may help address serotype replacement and further reduce disease burden.

The importance of selecting appropriate first-line antibiotics cannot be overstated, especially given the high non-susceptibility rates in non-invasive pneumococcal strains. As compared with other commonly used antibiotics in the paediatric population, AMO resistance remains relatively low at 25.2%, and so it may be a first-line treatment option for IPD. The emergence of resistance underscores the need for continuous surveillance of antimicrobial efficacy and the implementation of robust antimicrobial stewardship programmes. With the prevalence of multidrug-resistant strains, such as GPSC1, considering alternative antibiotic options is crucial. However, treatment should not be the sole focus; prevention through vaccination and improved infection control practices must also play a central role. The introduction of the PCV13 vaccine to the NIP is likely to reduce *S. pneumoniae* associated antibiotic resistance (AMR) as nearly all isolates in this study are VTs. Additionally, vaccination is expected to reduce the number of diseases caused by *S. pneumoniae*, thereby decreasing the need for antibiotic prescriptions and reducing exposure to antibiotics.

International collaboration and data sharing are also vital in combating antibiotic resistance. By working together, countries can share best practices, monitor emerging resistance patterns and develop coordinated strategies to limit the spread of resistant strains. Public awareness campaigns to educate the public on responsible antibiotic use will also help reduce antibiotic misuse.

Despite the valuable insights provided by this study, several limitations should be acknowledged. First, as the study was conducted in a single region, its findings may not be fully generalizable to other areas with differing pneumococcal strain distributions or antibiotic resistance patterns. However, although this study is limited to Suzhou, similarly low levels of PCV13 uptake are predicted to be common across China [42]. Additionally, changes in the sampling strategy after 2018, which prioritized IPD cases, may have introduced bias by underrepresenting non-IPD strains in recent years, potentially affecting estimates of serotype prevalence and vaccine coverage. Lastly, while the study captures significant serotype and genetic diversity, it does not account for variations in disease severity or long-term clinical outcomes across different patient populations.

## Conclusion

The introduction of PCVs into the NIP, particularly PCV13 or higher valency vaccines (PCV15/PCV20), alongside enhanced antibiotic stewardship and infection prevention strategies, presents a clear pathway to reducing the burden of pneumococcal disease and combating antibiotic resistance in China. This comprehensive approach will improve child health, mitigate the risks associated with drug-resistant *S. pneumoniae* and contribute to global efforts in fighting antibiotic resistance.

## Supplementary material

10.1099/mgen.0.001398 Supplementary Material 1.

10.1099/mgen.0.001398 Supplementary Material 2.

10.1099/mgen.0.001398 Supplementary Material 3.

10.1099/mgen.0.001398 Uncited Supplementary Data Sheet 1.

## Abbreviations

AMO: amoxicillin
AMR: antimicrobial resistance
CFT: ceftriaxone
CFX: cefuroxime
CHL: chloramphenicol
CLI: clindamycin
COT: cotrimoxazole
DOX: doxycycline
ERY: erythromycin
GPSCs: Global Pneumococcal Sequence Clusters
IPD: invasive pneumococcal disease
MER: meropenem
NIP: National Immunization Program
PCV10: 10-valent PCV
PCV13: 13-valent PCV
PCV: pneumococcal conjugate vaccine
PEN: penicillin
TAX: cefotaxime
TET: tetracycline
VTs: vaccine serotypes
WGS: whole-genome sequencing
