# Supplementary material for: Genomic analysis of invasive and non-invasive disease-causing Streptococcus pneumoniae isolated from children between 2014 and 2023 in Suzhou, China
Source: Microb Genom. 2025 Jun 2;11(6):001398. doi: 10.1099/mgen.0.001398 (PMC12130468; doi:10.1099/mgen.0.001398)
Supplement: Supplementary Material 1. [file mgen-11-01398-s001.pdf]

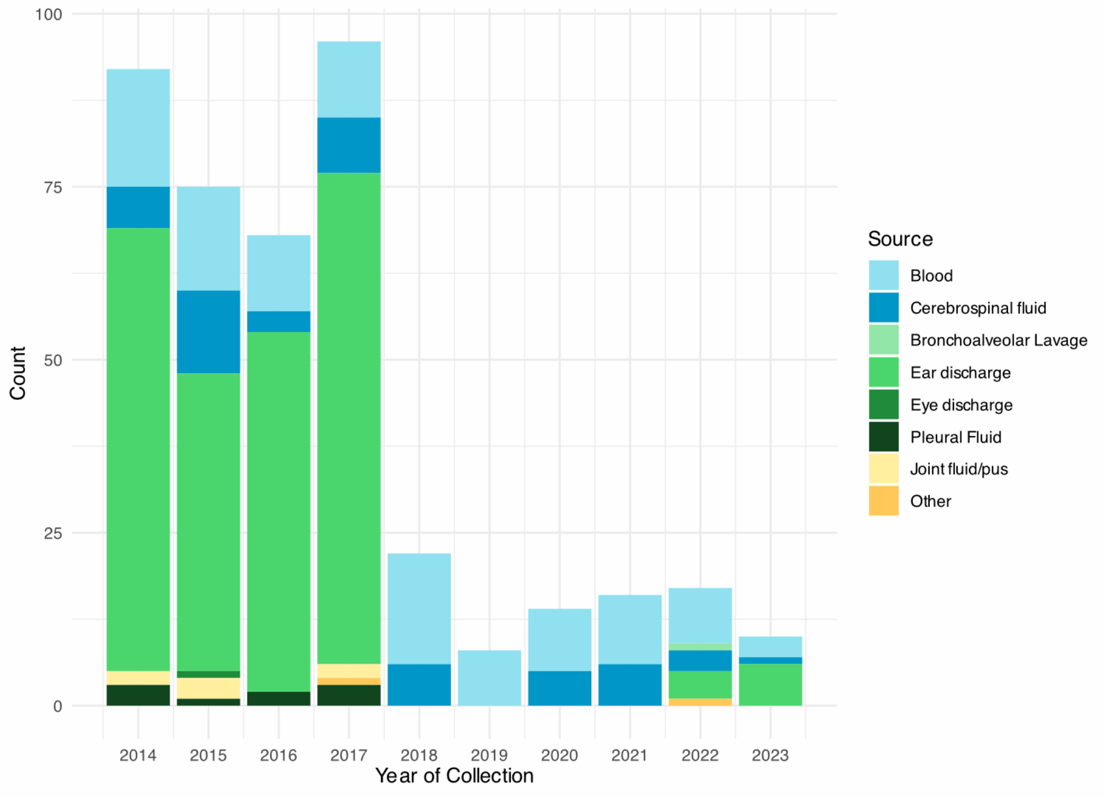

Figure S1 – Count of isolates collected each year, coloured by the source of the isolate. Shades of green show non-IPD collections, blue show IPD collections, and yellow show cases where it is unclear. After 2018, the strategy for collection changed to focus on IPD, leading to fewer total isolates being collected per year, and a big decrease in non-IPD samples.
